# Muscle phosphorus metabolites in sarcopenia

**DOI:** 10.18632/aging.104032

**Published:** 2020-08-28

**Authors:** J. Matthew Hinkley, Paul M. Coen

**Affiliations:** 1 AdventHealth Translational Research Institute, Orlando, FL 32804, USA

**Keywords:** aging, muscle, sarcopenia, phosphodiester, phosphocreatine

Phosphorus is a macromineral with numerous important biologic functions. Metabolites with a phosphorus moiety include structural lipids of the cell membrane (phospholipids) and nucleic acid molecules. Phosphorus metabolites also play an important role in metabolic activity and energy metabolism, processes that inherently depend on the capacity to phosphorylate intermediate metabolites and to store energy released during oxidative phosphorylation in high-energy phosphate bonds such as ATP or phosphocreatine. In skeletal muscle, phosphorus metabolites are essential for providing high-energy phosphates for contractile activity and structural integrity of the sarcolemma membrane and intracellular organelles including the sarcoplasmic and mitochondrial reticulum.

The age-related loss of skeletal muscle mass and function (sarcopenia) is a significant healthcare problem for older adults and an unmet medical need. The use of 31-phosphorus magnetic resonance spectroscopy (^31^P-MRS) is a valuable tool to assess *in vivo* phosphorus metabolites and has been used to further understand skeletal muscle aging. While the phosphocreatine peak is most prominent in the spectra, several additional peaks (e.g., phosphodiester (PDE) 1 and 2, putative readouts of membrane damage) can be quantified by obtaining the fully relaxed spectrum over a longer (12 minute) acquisition time to improve spectral resolution. This is important, as recent work has shown that resting levels of phosphocreatine and PDE in skeletal muscle appear be linked with whole-body and skeletal muscle metabolism including insulin sensitivity, glucose homeostasis, and mitochondrial content [1]. We recently identified resting phosphorus metabolites that were associated with lower muscle mass and function in older adults [2]. In a group of 55 older obese adults, we observed that lower *in vivo* phosphocreatine content was associated with lower muscle volume and knee extensor peak power, while an elevated PDE2 peak was negatively related to these variables. We further validated these findings in a second group of well-phenotyped older adults classified as non-sarcopenic or sarcopenic based on the European Working Group on Sarcopenia in Older People (EWGSOP) definition and observed that phosphocreatine was lower in muscle from sarcopenic adults while PDE2 was higher.

Phosphocreatine is a crucial energy source in metabolically demanding tissues (i.e., skeletal muscle and brain) [3]. In skeletal muscle, phosphocreatine provides a readily available energy source during short, high-intensity periods of contractile activity (for example during a loss of balance and to prevent a fall) by transferring the N-phosphoryl group of phosphocreatine to ADP to synthesize ATP [3]. Creatine supplementation has been shown to be an effective intervention to increase phosphocreatine stores in metabolically demanding tissues, which may aid in important cellular functions with aging. Specifically, creatine supplementation alone can enhance upper and lower body strength in older adults, along with increases in total and lean body mass [4,5]. Further, in conjunction with resistance exercise training, creatine supplementation can promote greater improvements in skeletal muscle function compared to exercise alone [3,6]. The lower level of phosphocreatine in sarcopenic muscle observed in our recent study further define the role of the creatine/phosphocreatine system on skeletal muscle health and are in-line with literature which suggests that creatine supplementation may be an important therapeutic intervention to improve muscle mass and strength in aging.

Cellular membranes consist primarily of phospholipids, including phosphatidylcholine (PC) and phosphatidylethanolamine (PE). Along with maintaining structure, the phospholipid composition of cellular membranes can impact several biological functions important to skeletal muscle health, including controlling intracellular calcium levels and mitochondrial energetics [7]. Given the observation of elevated PDE2, we performed a lipidomics analysis on biopsy specimens from the vastus lateralis to examine skeletal muscle phospholipids. Older adults with sarcopenia had elevated levels of PC and PE in comparison to non-sarcopenic counterparts and heightened levels of these phospholipids were negatively associated with muscle volume and peak knee extensor power [2]. These data are similar to recent pre-clinical work by Uchitomi et al., who observed an increase in phospholipid levels in aged mouse muscle which was correlated with lower fast-twitch muscle fiber size, a hallmark of sarcopenia [8]. Further, our findings support a growing consensus that, beyond its role on cellular structure, alterations in myocellular phospholipid composition appear to influence biological functions critical for maintaining skeletal muscle mass and function [7].

In order to successfully develop effective therapeutics to halt the progression of sarcopenia it is important to understand the underlying etiology to identify potential therapeutic targets. Our results suggest phosphorus metabolites involved in energy metabolism (phosphocreatine) and cellular membrane integrity (PDE and phospholipids) are distinct in sarcopenia and are related to lower muscle mass and function in older adults [2]. This adds to a growing consensus on the role of phosphorus metabolites on whole-body and skeletal muscle metabolic health [1,2]. Whether the changes in phosphorus metabolites are a causal factor in mediating impaired metabolic function and loss of muscle mass and function or just a consequence of the aging phenotype is unknown. Longitudinal studies are needed to establish causation or to determine if baseline phosphorus metabolites in skeletal muscle can predict whether older adults are more prone to develop the sarcopenia or other metabolic disorders (i.e., type 2 diabetes).
